# New Developments in Statistical Information Theory Based on Entropy and Divergence Measures

**DOI:** 10.3390/e21040391

**Published:** 2019-04-11

**Authors:** Leandro Pardo

**Affiliations:** Department of Satistics and Operation Research, Faculty of Mathematics, Universidad Complutense de Madrid, 28040 Madrid, Spain; lpardo@mat.ucm.es

In the last decades the interest in statistical methods based on information measures and particularly in pseudodistances or divergences has grown substantially. Minimization of a suitable pseudodistance or divergence measure gives estimators (minimum pseudodistance estimators or minimum divergence estimators) that have nice robustness properties in relation to the classical maximum likelihood estimators with a not significant lost of efficiency. For more details we refer the monographs of Basu et al. [1] and Pardo [2]. Parametric test statistics based on the minimum divergence estimators have also given interesting results in relation to the robustness in comparison with the classical likelihood ratio test, Wald test statistic and Rao’s score statistic. Worthy of special mention are the Wald-type test statistics obtained as an extension of the classical Wald test statistic. These test statistics are based on minimum divergence estimators instead of the maximum likelihood estimators and have been considered in many different statistical problems: Censoring, see Ghosh et al. [3], equality of means in normal and lognormal models, see Basu et al. [4,5], logistic regression models, see Basu et al. [6], polytomous logistic regression models, see Castilla et al. [7], composite likelihood methods, see Martín et al. [8], etc.

This Special Issue focuses on original and new research based on minimum divergence estimators, divergence statistics as well as parametric tests based on pseudodistances or divergences, from a theoretical and applied point of view, in different statistical problems with special emphasis on efficiency and robustness. It comprises 15 selected papers that address novel issues, as well as specific topics illustrating the importance of the divergence measures or pseudodistances in statistics. In the following, the manuscripts are presented in alphabetical order.

The paper, “A Generalized Relative (α,β)-Entropy Geometric properties and Applications to Robust Statistical Inference”, by A. Ghosh and A. Basu [9], proposes an alternative information theoretic formulation of the logarithmic super divergence (LSD), Magie et al. [10], as a two parametric generalization of the relative α—entropy, which they refer as the general (α,β)-entropy. The paper explores its relation with various other entropies and divergences, which also generates a two-parameter extension of Renyi entropy measure as a by-product. The paper is primarily focused on the geometric properties of the relative (α,β)-entropy or the LSD measures: Continuity and convexity in both the arguments along with an extended Pythagorean relation under a power-transformation of the domain space. They also derived a set of sufficient conditions under which the forward and the reverse projections of the relative (α,β)-entropy exist and are unique. Finally, they briefly discuss the potential applications of the relative (α,β)-entropy or the LSD measures in statistical inference, in particular, for robust parameter estimation and hypothesis testing. The results in the reverse projection of the relative (α,β)-entropy establish, for the first time, the existence and uniqueness of the minimum LSD estimators. Numerical illustrations are also provided for the problem of estimating the binomial parameter.

In the work “Asymptotic Properties for methods Combining the Minimum Hellinger Distance Estimate and the Bayesian Nonparametric Density Estimate”, Wu, Y. and Hooker, G. [11], pointed out that in frequentist inference, minimizing the Hellinger distance (Beran et al. [12]) between a kernel density estimate and a parametric family produces estimators that are both robust to outliers and statistically efficient when the parametric family contains the data-generating distribution. In this paper the previous results are extended to the use of nonparametric Bayesian density estimators within disparity methods. They proposed two estimators: One replaces the kernel density estimator with the expected posterior density using a random histogram prior; the other transforms the posterior over densities into a posterior over parameters through minimizing the Hellinger distance for each density. They show that it is possible to adapt the mathematical machinery of efficient influence functions from semiparametric models to demonstrate that both estimators introduced in this paper are efficient in the sense of achieving the Cramér-Rao lower bound. They further demonstrate a Bernstein-von-Mises result for the second estimator, indicating that its posterior is asymptotically Gaussian. In addition, the robustness properties of classical minimum Hellinger distance estimators continue to hold.

In “Composite Likelihood Methods Based on Minimum Density Power Divergence Estimator”, E. Castilla, N. Martin, L. Pardo and K. Zografos [13] pointed out that the classical likelihood function requires exact specification of the probability density function, but in most applications, the true distribution is unknown. In some cases, where the data distribution is available in an analytic form, the likelihood function is still mathematically intractable due to the complexity of the probability density function. There are many alternatives to the classical likelihood function; in this paper, they focus on the composite likelihood. Composite likelihood is an inference function derived by multiplying a collection of component likelihoods; the particular collection used is a conditional determined by the context. Therefore, the composite likelihood reduces the computational complexity, so that it is possible to deal with large datasets and very complex models even when the use of standard likelihood methods is not feasible. Asymptotic normality of the composite maximum likelihood estimator (CMLE) still holds with the Godambe information matrix to replace the expected information in the expression of the asymptotic variance-covariance matrix. This allows the construction of composite likelihood ratio test statistics, Wald-type test statistics, as well as score-type statistics. A review of composite likelihood methods is given in Varin [14]. They mentioned at this point that CMLE, as well as the respective test statistics are seriously affected by the presence of outliers in the set of available data. The main purpose of this paper is to introduce a new robust family of estimators, namely, composite minimum density power divergence estimators (CMDPDE), as well as a new family of Wald-type test statistics based on the CMDPDE in order to get broad classes of robust estimators and test statistics. A simulation study is presented, in order to study the robustness of the CMDPDE, as well as the performance of the Wald-type test statistics based on CMDPDE.

The paper “Composite Tests under Corrupted Data”, by M. Broniatowski, J. Jurecková, A. Kumar Moses and E. Miranda [15] investigate test procedures under corrupted data. They assume that the observations $Z_{i}$ are mismeasured, due to the presence of measurement errors. Thus, instead of observing $Z_{i}$ for i=1,…,n, we observe $X_{i}=Z_{i}+\sqrt{\delta }V_{i},$ with an unknown parameter δ and an unobservable random variable $V_{i}$. It is assumed that the random variables $Z_{i}$ are independent and identically distributed, as are the $X_{i}$ and the $V_{i}$. The test procedure aims at deciding between two simple hypotheses pertaining to the density of the variable $Z_{i}$, namely $f_{0}$ and $g_{0}$. In this setting, the density of the $V_{i}$ is supposed to be known. The procedure which they propose aggregates likelihood ratios for a collection of values of δ. A new definition of least-favorable hypotheses for the aggregate family of tests is presented, and a relation with the Kullback-Leibler divergence between the sets $f_{\delta }\left(\delta \right)$ and $g_{\delta }\left(\delta \right)$ is presented. Finite-sample lower bounds for the power of these tests are presented, both through analytical inequalities and through simulation under the least-favorable hypotheses. Since no optimality holds for the aggregation of likelihood ratio tests, a similar procedure is proposed, replacing the individual likelihood ratio by some divergence based test statistics. It is shown and discussed that the resulting aggregated test may perform better than the aggregate likelihood ratio procedure.

The article “Convex Optimization via Symmetrical Hölder Divergence for a WLAN Indoor Positioning System”, by O. Abdullah [16], uses the Hölder divergence, which generalizes the idea of divergence in information geometry by smooth the non-metric of statistical distances in a way that are not required to follow the law of indiscernibles. The inequality of log-ratio gap pseudo-divergence is built to measure the statistical distance of two classes based on Hölder’s ordinary divergence. By experiment, the WiFi signal suffers from multimodal distribution; nevertheless, the Hölder divergence is considered the proper divergence to measure the dissimilarities between probability densities since the Hölder divergence is a projective divergence that does not need the distribution be normalized and allows the closed form expressions when the expansion family is an affine natural space like multinomial distributions. Hölder divergences encompass both the skew Bhattacharyya divergences and Cauchy-Schwarz divergence, Nielsen et al. [17], and can be symmetrized, and the symmetrized Hölder divergence outperformed the symmetrized Cauchy-Schwarz divergence over the dataset of Gaussians. Both Cauchy-Schwarz divergences are part of a projective divergence distance family with a closed-form expression that does not need to be normalized when considering closed-form expressions with an affine and conic parameter space, such as multivariate or multinomial distributions.

In the paper “Likelihood Ratio Testing under Measurement Errors”, M. Broniatowski, J. Jurecková and J. Kalina [18] consider the likelihood ratio test of a simple null hypothesis (with density $f_{0}$) against a simple alternative hypothesis (with density $g_{0}$) in the situation that observations $X_{i}$ are mismeasured due to the presence of measurement errors. Thus instead of $X_{i}$ for i=1,…,n, we observe $Z_{i}=X_{i}+\sqrt{\delta }V_{i}$ with unobservable parameter δ and unobservable random variable $V_{i}.$ When we ignore the presence of measurement errors and perform the original test, the probability of type I error becomes different from the nominal value, but the test is still the most powerful among all tests on the modified level. Further, they derive the minimax test of some families of misspecified hypotheses and alternatives.

The paper “Minimum Penalized ϕ-Divergence Estimation under Model Misspecification”, by M. V. Alba-Fernández, M. D. Jiménez-Gamero and F. J. Ariza-López [19], focuses on the consequences of assuming a wrong model for multinomial data when using minimum penalized ϕ-divergence, also known as minimum penalized disparity estimators, to estimate the model parameters. These estimators are shown to converge to a well-defined limit. An application of the results obtained shows that a parametric bootstrap consistently estimates the null distribution of a certain class of test statistics for model misspecification detection. An illustrative application to the accuracy assessment of the thematic quality in a global land cover map is included.

In “Non-Quadratic Distances in Model Assessment”, M. Markatou and Y. Chen [20] consider that as a natural way to measure model adequacy is by using statistical distances as loss functions. A related fundamental question is how to construct loss functions that are scientifically and statistically meaningful. In this paper, they investigate non-quadratic distances and their role in assessing the adequacy of a model and/or ability to perform model selection. They first present the definition of a statistical distance and its associated properties. Three popular distances, total variation, the mixture index of fit and the Kullback-Leibler distance, are studied in detail, with the aim of understanding their properties and potential interpretations that can offer insight into their performance as measures of model misspecification. A small simulation study exemplifies the performance of these measures and their application to different scientific fields is briefly discussed.

In “ϕ-Divergence in Contingency Table Analysis”, M. Kateri [21] presents a review about the role of ϕ-divergence measures, see Pardo [2], in modelling association in two-way contingency tables, and illustrated it for the special case of uniform association in ordinal contingency tables. This is targeted at pointing out the potential of this modelling approach and the generated families of models. Throughout this paper a multinomial sampling scheme is assumed. For the models considered here, the other two classical sampling schemes for contingency tables (independent Poisson and product multinomial) are inferentially equivalent. Furthermore, for ease of presentation, we restricted here to two-way tables. The proposed models extend straightforwardly to multi-way tables. For two or higher-dimensional tables, the subset of models that are linear in their parameters (i.e., multiplicative Row-Column (RC) and RC(M)-type terms are excluded) belong to the family of homogeneous linear predictor models, Goodman [22] and can thus be fitted using the R-package *mph*.

In “Robust and Sparse Regression via γ-Divergence”, T. Kawashima and H. Fujisawa [23] study robust and sparse regression based on the γ-divergence. They showed desirable robust properties under both homogeneous and heterogeneous contamination. In particular, they presented the Pythagorean relation for the regression case, although it was not shown in Kanamori and Fujisawa, [24]. In most of the robust and sparse regression methods, it is difficult to obtain the efficient estimation algorithm, because the objective function is non-convex and non-differentiable. Nonetheless, they succeeded to propose the efficient estimation algorithm, which has a monotone decreasing property of the objective function by using the Majorization–Minimization algorithm (MM-algorithm). The numerical experiments and real data analyses suggested that their method was superior to comparative robust and sparse linear regression methods in terms of both accuracy and computational costs. However, in numerical experiments, a few results of performance measure “true negative rate (TNR)” were a little less than the best results. Therefore, if more sparsity of coefficients is needed, other sparse penalties, e.g., the Smoothly Clipped Absolute Deviations (SCAD), see Fan et al. [25] and the Minimax Concave Penalty (MCP), see Zhang [26], can also be useful.

The manuscript “Robust-Bregman Divergence (BD) Estimation and Inference for General Partially Linear Models”, by C. Zhang and Z. Zhang [27], proposes a class of “robust-Bregman divergence (BD)” estimators of both the parametric and nonparametric components in the general partially linear model (GPLM), which allows the distribution of the response variable to be partially specified, without being fully known. Using the local-polynomial function estimation method, they proposed a computationally-efficient procedure for obtaining “robust-BD” estimators and established the consistency and asymptotic normality of the “robust-BD” estimator of the parametric component $\beta_{0}$. For inference procedures of $\beta_{0}$ in the GPLM, they show that the Wald-type test statistic, $W_{n}$, constructed from the “robust-BD” estimators is asymptotically distribution free under the null, whereas the likelihood ratio-type test statistic, $\Lambda_{n},$ is not. This provides an insight into the distinction from the asymptotic equivalence (Fan and Huang, [28]) between $W_{n}$ and $\Lambda_{n}$ in the partially linear model constructed from profile least-squares estimators using the non-robust quadratic loss. Numerical examples illustrate the computational effectiveness of the proposed “robust-BD” estimators and robust Wald-type test in the appearance of outlying observations.

In “Robust Estimation for the Single Index Model Using Pseudodistances”, A. Toma and C. Fulga [29] consider minimum pseudodistance estimators for the parameters of the single index model (model to reduce the number of parameters in portfolios), see Sharpe [30], and using them they construct new robust optimal portfolios. When outliers or atypical observations are present in the data set, the new portfolio optimization method based on robust minimum pseudodistance estimates yields better results than the classical single index method based on maximum likelihood estimates, in the sense that it leads to larger returns for smaller risks. In literature, there exist various methods for robust estimation in regression models. In the present paper, they proposed the method based on the minimum pseudodistance approach, which suppose to solve a simple optimization problem. In addition, from a theoretical point of view, these estimators have attractive properties, such as being redescending robust, consistent, equivariant and asymptotically normally distributed. The comparison with other known robust estimators of the regression parameters, such as the least median of squares estimators, the S-estimators or the minimum density power divergence estimators, shows that the minimum pseudodistance estimators represent an attractive alternative that may be considered in other applications too. They study properties of the estimators, such as, consistency, asymptotic normality, robustness and equivariance and illustrate the benefits of the proposed portfolio optimization method through examples for real financial data.

The paper “Robust Inference after Random Projections via Hellinger Distance for Location-scale Family”, by L. Li, A. N. Vidyashankar, G. Diao and E. Ahmed [31], proposes Hellinger distance based methods to obtain robust estimates for mean and variance in a location-scale model that takes into account (i) storage issues, (ii) potential model misspecifications, and (iii) presence of aberrant outliers. These issues-which are more likely to occur when dealing with massive amounts of data-if not appropriately accounted in the methodological development, can lead to inaccurate inference and misleading conclusions. On the other hand, incorporating them in the existing methodology may not be feasible due to a computational burden. Our extensive simulations show the usefulness of the methodology and hence can be applied in a variety of scientific settings. Several theoretical and practical questions concerning robustness in a big data setting arise.

The paper “Robustness Property of Robust-BD Wald-Type Test for Varying-Dimensional General Linear Models” by X. Guo and C. Zhang [32], aims to demonstrate the robustness property of the robust-BD Wald-type test in Zhang et al. [33]. Nevertheless, it is a nontrivial task to address this issue. Although the local stability for the Wald-type tests have been established for the M-estimators, see Heritier and Ronchetti, [34], generalized method of moment estimators, Ronchetti and Trojan, [35], minimum density power divergence estimator, Basu et al. [36] and general M-estimators under random censoring, Ghosh et al. [3], their results for finite-dimensional settings are not directly applicable to our situations with a diverging number of parameters. Under certain regularity conditions, we provide rigorous theoretical derivations for robust testing based on the Wald-type test statistics. The essential results are approximations of the asymptotic level and power under contaminated distributions of the data in a small neighborhood of the null and alternative hypotheses, respectively.

The manuscript “Robust Relative Error Estimation” by K. Hirose and H. Masuda [37], presents a relative error estimation procedure that is robust against outliers. The proposed procedure is based on the γ-likelihood function, which is constructed by γ-cross entropy, Fujisawa and Eguch, [38]. They showed that the proposed method has the redescending property, a desirable property in robust statistics literature. The asymptotic normality of the corresponding estimator together with a simple consistent estimator of the asymptotic covariance matrix are derived, which allows the construction of approximate confidence sets. Besides the theoretical results, they have constructed an efficient algorithm, in which we minimize a convex loss function at each iteration. The proposed algorithm monotonically decreases the objective function at each iteration.

## References

[1] Basu A., Shioya H., Park C. (2011). Statistical Inference: The Minimum Distance Approach.

[2] Pardo L. (2006). Statistical Inference Based on Divergence Measures.

[3] Ghosh A., Basu A., Pardo L. (2017). Robust Wald-type tests under random censoring. arXiv.

[4] Basu A., Mandal A., Martín N., Pardo L. (2019). A Robust Wald-Type Test for Testing the Equality of Two Means from Log-Normal Samples. Methodol. Comput. Appl. Probab..

[5] Basu A., Mandal A., Martin N., Pardo L. (2015). Robust tests for the equality of two normal means based on the density power divergence. Metrika.

[6] Basu A., Ghosh A., Mandal A., Martín N., Pardo L. (2017). A Wald-type test statistic for testing linear hypothesis in logistic regression models based on minimum density power divergence estimator. Electron. J. Stat..

[7] Castilla E., Ghosh A., Martín N., Pardo L. (2019). New robust statistical procedures for polytomous logistic regression models. Biometrics.

[8] Martín N., Pardo L., Zografos K. (2019). On divergence tests for composite hypotheses under composite likelihood. Stat. Pap..

[9] Ghosh A., Basu A. (2018). A Generalized Relative (*α*,*β*)-Entropy: Geometric Properties and Applications to Robust Statistical Inference. Entropy.

[10] Maji A., Ghosh A., Basu A. (2016). The Logarithmic Super Divergence and Asymptotic Inference Properties. AStA Adv. Stat. Anal..

[11] Wu Y., Hooker G. (2018). Asymptotic Properties for Methods Combining the Minimum Hellinger Distance Estimate and the Bayesian Nonparametric Density Estimate. Entropy.

[12] Beran R. (1977). Minimum Hellinger Distance Estimates for Parametric Models. Ann. Stat..

[13] Castilla E., Martín N., Pardo L., Zografos K. (2018). Composite Likelihood Methods Based on Minimum Density Power Divergence Estimator. Entropy.

[14] Varin C., Reid N., Firth D. (2011). An overview of composite likelihood methods. Stat. Sin..

[15] Broniatowski M., Jurečková J., Moses A.K., Miranda E. (2019). Composite Tests under Corrupted Data. Entropy.

[16] Abdullah O. (2018). Convex Optimization via Symmetrical Hölder Divergence for a WLAN Indoor Positioning System. Entropy.

[17] Nielsen F., Sun K., Marchand-Maillet S. k-Means Clustering with Hölder Divergences. Proceedings of the International Conference on Geometric Science of Information.

[18] Broniatowski M., Jurečková J., Kalina J. (2018). Likelihood Ratio Testing under Measurement Errors. Entropy.

[19] Alba-Fernández M.V., Jiménez-Gamero M.D., Ariza-López F.J. (2018). Minimum Penalized *ϕ*-Divergence Estimation under Model Misspecification. Entropy.

[20] Markatou M., Chen Y. (2018). Non-Quadratic Distances in Model Assessment. Entropy.

[21] Kateri M. (2018). *ϕ*-Divergence in Contingency Table Analysis. Entropy.

[22] Goodman L.A. (1981). Association models and canonical correlation in the analysis of cross-classifications having ordered categories. J. Am. Stat. Assoc..

[23] Kawashima T., Fujisawa H. (2017). Robust and Sparse Regression via *γ*-Divergence. Entropy.

[24] Kanamori T., Fujisawa H. (2015). Robust estimation under heavy contamination using unnormalized models. Biometrika.

[25] Fan J., Li R. (2001). Variable Selection via Nonconcave Penalized Likelihood and its Oracle Properties. J. Am. Stat. Assoc..

[26] Zhang C.H. (2010). Nearly unbiased variable selection under minimax concave penalty. Ann. Stat..

[27] Zhang C., Zhang Z. (2017). Robust-BD Estimation and Inference for General Partially Linear Models. Entropy.

[28] Fan J., Huang T. (2005). Profile likelihood inferences on semiparametric varying-coefficient partially linear models. Bernoulli.

[29] Toma A., Fulga C. (2018). Robust Estimation for the Single Index Model Using Pseudodistances. Entropy.

[30] Sharpe W.F. (1963). A simplified model to portfolio analysis. Manag. Sci..

[31] Li L., Vidyashankar A.N., Diao G., Ahmed E. (2019). Robust Inference after Random Projections via Hellinger Distance for Location-scale Family. Entropy.

[32] Guo X., Zhang C. (2018). Robustness Property of Robust-BD Wald-Type Test for Varying-Dimensional General Linear Models. Entropy.

[33] Zhang C.M., Guo X., Cheng C., Zhang Z.J. (2012). Robust-BD estimation and inference for varying-dimensional general linear models. Stat. Sin..

[34] Heritier S., Ronchetti E. (1994). Robust bounded-influence tests in general parametric models. J. Am. Stat. Assoc..

[35] Ronchetti E., Trojani F. (2001). Robust inference with GMM estimators. J. Econom..

[36] Basu A., Ghosh A., Martin N., Pardo L. (2018). Robust Wald-type tests for non-homogeneous observations based on minimum density power divergence estimator. Metrika.

[37] Hirose K., Masuda H. (2018). Robust Relative Error Estimation. Entropy.

[38] Fujisawa H., Eguchi S. (2008). Robust parameter estimation with a small bias against heavy contamination. J. Multivar. Anal..

